# The Influencing Legal and Factors of Migrant Children’s Educational Integration Based on Convolutional Neural Network

**DOI:** 10.3389/fpsyg.2021.762416

**Published:** 2022-01-10

**Authors:** Chi Zhang, Gang Wang, Jinfeng Zhou, Zhen Chen

**Affiliations:** ^1^School of Marxism, Northeast Forestry University, Harbin, China; ^2^China Biodiversity Conservation and Green Development Foundation, Beijing, China; ^3^School of Marxism, Northeastern University, Shenyang, China

**Keywords:** convolutional neural network algorithm, attention mechanism, text classification model, migrant children, educational integration

## Abstract

This research aims to analyze the influencing factors of migrant children’s education integration based on the convolutional neural network (CNN) algorithm. The attention mechanism, LSTM, and GRU are introduced based on the CNN algorithm, to establish an ALGCNN model for text classification. Film and television review data set (MR), Stanford sentiment data set (SST), and news opinion data set (MPQA) are used to analyze the classification accuracy, loss value, Hamming loss (HL), precision (Pre), recall (Re), and micro-F1 (F1) of the ALGCNN model. Then, on the big data platform, data in the *Comprehensive Management System of Floating Population and Rental Housing*, *Student Status Information Management System*, and *Student Information Management System* of Beijing city are taken as samples. The ALGCNN model is used to classify and compare related data. It is found that in the MR, STT, and MPQA data sets, the classification accuracy and loss value of the ALGCNN model are better than other algorithms. HL is the lowest (15.2 ± 1.38%), the *Pre* is second only to the BERT algorithm, and the Re and F1 are both higher than other algorithms. From 2015 to 2019, the number of migrant children in different grades of elementary school shows a gradual increase. Among migrant children, the number of migrant children from other counties in this province is evidently higher than the number of migrant children from other provinces. Among children of migrant workers, the number of immigrants from other counties in this province is also notably higher than the number of immigrants from other provinces. With the gradual increase in the years, the proportion of township-level expenses shows a gradual decrease, whereas the proportion of district and county-level expenses shows a gradual increase. Moreover, the accuracy of the ALGCNN model in migrant children and local children data classification is 98.6 and 98.9%, respectively. The proportion of migrant children in the first and second grades of a primary school in Beijing city is obviously higher than that of local children (*p* < 0.05). The average final score of local children was greatly higher than that of migrant children (*p* < 0.05), whereas the scores of migrant children’s listening methods, learning skills, and learning environment adaptability are lower, which shows that an effective text classification model (ALGCNN) is established based on the CNN algorithm. In short, the children’s education costs, listening methods, learning skills, and learning environment adaptability are the main factors affecting migrant children’s educational integration, and this work provides a reference for the analysis of migrant children’s educational integration.

## Introduction

Since the reform and opening-up, the scale floating population in China has shown a trend of increasing year by year (Wang et al., 2019). After 1990, “family” migration had developed in China’s migrant population, so that many school-age children followed their parents into cities, and the number increased dramatically (Ajay et al., 2018). Childhood is an important period of education and physical and mental development, and children’s education relates to the harmony and stability of the entire society. To guarantee the education of migrant children, most national and local governments have successively promulgated relevant policies to give more support to migrant children during the compulsory education stage (Hu et al., 2018; Liang et al., 2019). The children migrating to the city have undergone a transformation of their living space to a certain extent. It should focus on whether the school’s learning environment can provide these migrant children with a comfortable and suitable education and living environment (Vogel, 2018), thereby eliminating or reducing the negative impacts on migrant children when they attend a school in the city with disadvantages in their families, schools, and society, and an unequal educational starting point. The level of education of migrant children and their integration with the cities where they flowed into not only have an important impact on China’s social stability and social development, but are also closely related to industrialization, urbanization, and the progression of the future labor market (Flensner et al., 2021). The direction of national education policy formulation changes with the change of social development. As a provider of public education services, the government has introduced various educational policies in the process of overall planning for the education of school-age migrant children, which will inevitably have a certain impact on the social integration of migrant children (Vargas-Valle and Aguilar-Zepeda, 2020). Scholars have been paying attention to the issue of migrant children’s social integration for a long time, but most of them start from the family environment or community level participation of migrant children (Kyereko and Faas, 2021). When it comes to the educational integration of migrant children, most studies are conducted from the perspective of school adaptation and the implementation of educational policies (Wang, 2020; Jørgensen et al., 2021), where those who adopt educational policy as an influencing factor to analyze the educational integration of migrant children are relatively rare.

Using big data to analyze the current situation of migrant children’s education is of great significance for us to correctly understand the current education status and future trends. When faced with big data, choosing an algorithm that can quickly and automatically extract text information is of great significance for improving the mining of effective information in the data. The vectorization result of the text directly determines the performance of the final classification of the text to a large extent (Lima et al., 2020). Text classification methods based on machine learning, such as support vector machine (SVM), decision tree (DT), and naive Bayes (NB), are proved to be suitable for effective classification of text information (Liang and Yi, 2021). Logistic regression and Softmax in the deep learning algorithm can also be applied to the feature extraction and classification of text information, and the classification effect is greatly superior to that of the machine learning algorithm (Griffiths and Boehm, 2019). However, these models face more complex problems due to factors such as human interference and training abnormalities, which leads to their classification results to be further optimized. Convolutional neural network (CNN) obtains local features through the movement of the window and shows the highest usage rate in the form of maximum pooling and average pooling (Barberá et al., 2021). The long–short-term memory (LSTM) model overcomes the gradient disappearance through gate control. Some researchers proposed the gated recurrent unit (GRU) model, which can adaptively capture the dependence of different time scales through the recurrent block, and the attention mechanism is introduced to ensure the different effects of input data on output data (Dashtipour et al., 2020). Basiri et al. (2021) established an ABCNN model based on the CNN and attention mechanism, which improved the effective classification of text. However, different text classification models still have the influence of noise such as human factors during feature selection, which needs to be further optimized.

To sum up, studies adopting educational policy as an influencing factor to analyze the educational integration of migrant children are relatively rare. Therefore, attention mechanism, LSTM, and GRU are introduced based on the CNN algorithm, and an ALGCNN model for text classification is innovatively established. ALGCNN model classification performance is evaluated based on known public data sets, to analyze the big data of migrant children in China and the current situation of migrant children’s education in a primary school in Beijing city. The data in the *Comprehensive Management System of Floating Population and Rental Housing*, *Student Status Information Management System*, and *Student Information Management System* of Beijing city are taken as samples. The ALGCNN model is used to classify and analyze the samples to explore the relevant factors affecting education integration, to provide help for migrant children to establish better education integration, and to provide a basis for improving the education environment and policies of migrant children. This work also aims to provide research reference of relevant issues for the future legislative work, to ensure that the migrant students can get legal and effective guarantee mechanism as protection. It also provides more in-depth and more direct legal provisions for different students to receive fair education after indicating the direction and content.

## Methodology

### Definitions of Related Concepts

Migrant children: “children” refers to people in the childhood stage, and “migrant children” is a relatively special part of the “children” (Stadelmannsteffen, 2018). With the deepening of China’s urbanization process, more and more rural people choose to work in cities. As they migrate between cities and rural areas, a special group of “migrant children” is evolved (Schrier et al., 2019). Based on the *Education Policies for Migrant Children (Provisional)* promulgated by the State Education Commission of the People’s Republic of China (PRC) and the Ministry of Public Security of PRC in 1998, “migrant children” are defined as children who can learn in 6–14 years old (or 7–15 years old) and live with parents or other guardians in the inflow place temporarily for more than 6 months. Based on this concept, 6–14 years of age (7–15 years of age) is defined as the selection range of childhood, and the children who migrate to this city with parents or other guardians are included, whereas children who do not migrant with their parents or guardians (i.e., left-behind children) are excluded.

Social integration: social integration is based on pluralism. When the culture of the place of migration is relatively inclusive, new immigrants are more inclined to maintain the original cultural value, and their identities and values are reshaped in the new settlement place, which help the formation of a diversified social and economic order, so that all members of society can enjoy fair and equal rights (Carolin et al., 2018; Moksony and Hegedus, 2018). Social integration covers cultural, economic, social, and psychological integration, and integration emphasizes the suitable construction of different groups with new cultural meanings (Noyens et al., 2018). Based on this, social integration is defined to build a good and harmonious education environment under the cooperation and support of society, school, and family, to promote the education development of migrant children. In addition, social integration is not to assimilate children into urban children but to build an educational environment suitable for migrant children. Therefore, the social integration of migrant children is defined as the action and process for good education and life of migrant children in the school through the mutual influences among education system, teaching curriculum, teachers, parents, students, and migrant children.

### Construction of the Text Classification Model Base on Improved Conventional Neural Network

**FIGURE 1:**
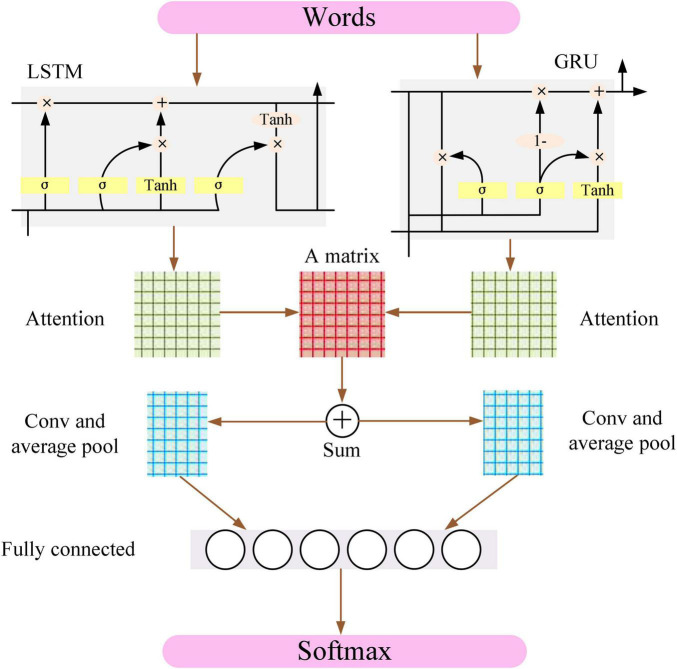
Basic structure of ALGCNN model.

The CNN model can capture local features such as keywords and phrases in sentences, but the most imperative step in the CNN model is how to determine the size of the convolution kernel (Chen and Jahanshahi, 2018). Recurrent neural network (RNN), LSTM, and GRU models can better grasp the features in the text and find the optimal feature (Iso et al., 2018; Jujie and Yaning, 2018). The data of the input model do not affect the output result because of the introduction of attention into the neural network model, and thus, it can optimize the text feature vector and improve the classification effect. Therefore, the LSTM model and the GRU model are combined in parallel based on the attention mechanism in the CNN model, and a new classification model ALGCNN is proposed, whose basic structure is shown in Figure 1.

The input layer of the ALGCNN model is composed of different words, and the dimension of each word is 286, so each sentence is expressed as a feature map of d*l (*l* is the length of the sentence).

**FIGURE 2:**
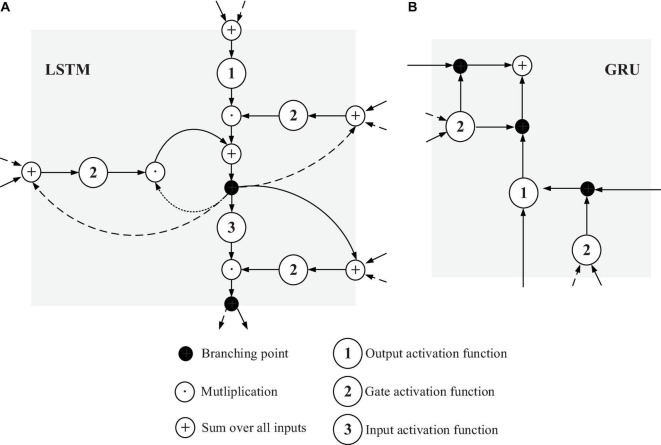
Basic structures of LSTM **(A)** and GRU **(B)**.

The LSTM in the network layer of LSTM and GRU is a variant of the RNN model and is mainly used to overcome gradient disappearance in the model. GRU model performs the simplification of LSTM. The basic structures of LSTM and GRU are illustrated in Figure 2 below.

Figure 2A shows that LSTM includes an input gate, a forget gate, an output gate, and a storage unit. Different gate structures control the input, storage, and output of the data stream. In LSTM, if the matrix *x*_*t*_ at time *t* is given, the mathematical expression calculated by the forget gate can be written as below equation:


(1) $$f_{t}=\sigma \,\left(\omega_{f}\cdot \left[y_{t-1}⊕x_{t}\right]+b_{f}\right)$$

In the above equation, σ refers to the Sigmoid function, ω*_*f*_* is the weight matrix of the forget gate, *y*_*t*–1_ represents the output value at the previous moment, ⊕ is the splicing of different matrices, and *b*_*f*_ represents the deviation value of the forget gate.

The input gate is mainly responsible for the input of current position of the sequence, and the calculation process through the input gate is expressed as Equation (2) below.


(2) $$\left\{ \begin{array}{c} u_{t}=\sigma \,\left(\omega_{u}\cdot \left[y_{t-1}⊕x_{t}\right]+b_{u}\right)\\ v_{t}=\delta \,\left(\omega_{v}\cdot \left[y_{t-1}⊕x_{t}\right]+b_{v}\right)\\ c_{t}=f_{t}\cdot c_{t-1}+u_{t}\cdot v_{t} \end{array}\right.$$

In the above equation, *u*_*t*_ is the first step of the calculation (it must consider how much of the current input information needs to be stored in the storage unit), *v*_*t*_ is the second step of the calculation (how much new information in the control input needs to be stored), δ refers to the Tanh activation function, *c*_*t*_ is the update of the neuron, and *b* is the deviation value.

The output value obtained by the output gate can be transmitted to the next moment, and the calculation process of the output gate is given as below:


(3) $$\left\{ \begin{array}{c} z_{t}=\sigma \,\left(\omega_{z}\cdot \left[y_{t-1}⊕x_{t}\right]+b_{z}\right)\\ y_{t}=z_{t}\cdot \delta \,\left(c_{t}\right) \end{array}\right.$$

Figure 2B suggests that the GRU model does not contain a storage unit compared with the LSTM model. The calculation process of the GRU model is given in Equation (4).


(4) $$\left\{ \begin{array}{c} R_{t}=\sigma \,\left(\omega_{R}\cdot \left[y_{t-1}⊕x_{t}\right]\right)\\ T_{t}=\sigma \,\left(\omega_{T}\cdot \left[y_{t-1}⊕x_{t}\right]\right)\\ \bar{y_{t}}=\delta \,\left(\omega_{\bar{y}}\cdot \left[R_{t}⊗y_{t-1}⊕x_{t}\right]\right)\\ y_{t}=\left(1-T_{t}\right)\cdot y_{t-1}+T_{t}\cdot \bar{y_{t}} \end{array}\right.$$

In the above equation, *R*_*t*_ is the control unit of reset gate, *T*_*t*_ represents the control unit of update gate, and ⊗ is the multiplication of corresponding elements among the matrices.

Both the LSTM and GRU models in the constructed ALGCNN model are utilized to process the original text information, so the number of hidden neurons in the LSTM and GRU models is set to be the same.

Figure 1 shows that the constructed ALGCNN model contains the attention mechanism layer (Connie et al., 2018). After all elements are compared using the attention matrix A, the model will be overfitting due to too many parameters. Thus, values of all neurons in the model are summed in this article. If the output value of the LSTM and GRU model is *Q*_*model*_ ∈ ℜ*^n^*^⋅^*^s^*, then the model is ∈(LSTM, GRU). *n* in the equation refers to the number of hidden neurons in the LSTM and GRU model, and the matrix A can be calculated with below equation:


(5) $$A_{i,j}=E\,u\,c\,l\,i\,d\,e\,a\,n\,\left(Q_{L\,S\,T\,M}\,\left[:,i\right],Q_{G\,R\,U}\,\left[:,j\right]\right)$$

In the above Equation (5), *Euclidean ()* refers to the Euclidean distance, and it can be calculated with Equation (6).


(6) $$E\,u\,c\,l\,i\,d\,e\,a\,n\,\left(u,v\right)=\frac{1}{1+\left|u-v\right|}$$

The matrix A can be calculated with Equation (5) above, and then, the attention weight value *a*_*z*,*j*_ = Σ*A*[*j*,:] of the *j*th neuron in the LSTM output and the attention weight value *a*_1_*_,j_* = Σ*A*[:,*j*] of the *j*th neuron in the GRU output can be generated.

After the attention mechanism layer, the ALGCNN model can realize convolution and pooling operations. If *n* new features *c*_*n*_ are calculated from the convolution operation window on text feature *x*_*n*:_*_*n*_*_+_*_*w*_*_–1_, the calculation equation can be written as below:


(7) $$c_{i}=f\,\left(U\cdot x_{n:n+w-1}+b_{c}\right)$$

In the Equation (7) above, *b*_*c*_ refers to the deviation value, *f()* is a non-linear function, and the word window of the convolution kernel can generate a feature map *c* = *c*_1_⊕*c*_2_⊕,⋯,⊕*c*_*s*−*w* + 1_.

The constructed ALGCNN model can equalize the pooling operation based on the attention mechanism. The calculation equation of the pooling operation is given as below.


(8) $$Q_{i}^{p}\,\left[:,j\right]=\sum_{k=j:j+w}a_{i,k}\,c_{i}\,\left[:,k\right]$$

In the above equation, $Q_{i}^{p}\in {ℜ}^{n\cdot s_{i}}$, and *i* refers to LSTM or GRU model.

The last fully connected layer in the ALGCNN model is obtained by combining the results of LSTM and GRU pooling operations, and the fully connected layer is connected to the Softmax classifier with below equation.


(9) $$\left\{ \begin{array}{c} p^{∼}\,\left(y|s\right)=S\,o\,f\,t\,m\,a\,x\,\left(\omega_{s}\cdot N+b_{s}\right)\\ y^{∼}=\arg\,\max_{y}p^{∼}\,\left(y|s\right) \end{array}\right.$$

In the equation given above, *N* is the combined value of pooling results of LSTM and GRU, and *y*^∼^ is the possible label value through the Softmax classifier.

Loss function of the model is the crossentropy loss function, which can be calculated with below equation:


(10) $$S\,\left(\theta \right)=-\frac{1}{K}\,\sum_{k=1}^{K}y_{k}\,\log\left(y_{k}^{∼}\right)+\lambda \,{||\theta ||}^{2}$$

In the equation above, *K* is the text category, *y*_*k*_ is the true category label, *y*_*k*_^∼^ is the label estimated by Softmax, and λ is the regularized hyperparameter of L2.

Finally, the model is optimized with the batch gradient descent (Wu et al., 2019) and Adam algorithm (Caltagirone and Frincke, 2005).

### Model Evaluation Indicators

In this study, the model is evaluated regarding the Hamming loss (HL), precision (Pre), recall (Re), and micro-F1 (F1). HL is often used as an evaluation indicator for multilabel classification, which indicates the number of misclassifications and misclassifications of a label. The smaller it is, the better the performance of the model. The calculation method was given as follows:


(11) $$H\,L=\frac{1}{N}\,\frac{X\,O\,R\,\left(Y_{i,j},P_{i,j}\right)}{L}$$

In the equation above, *N* is the number of samples, *L* refers to the total number of labels, *Y*_*i,j*_ is the j-th label in the true label set of the i-th sample, *P*_*i*,*j*_ represents the j-th label in the predicted label set of the i-th sample, and *XOR* is an exclusive OR operation.

*Pre* represents the proportion of correct predictions in samples with positive predictions, and it reflects the precision of the model, which is calculated with below equation:


(12) $$\mathrm{Pr}e=\frac{T\,P}{T\,P+F\,P}$$

The *Re* indicates the proportion of the positive samples in the sample that are correctly predicted. It reflects the recall rate of the model, whose calculation method is expressed as follows. The greater the *Pre* and *Re*, the better the effect of the model.


(13) $$R\,e=\frac{T\,P}{T\,P+F\,N}$$

In Equations (12) and (13), *TP* represents the original number of positive samples predicted to be positive, *FP* represents the original number of positive samples predicted to be negative, and *FN* represents the original number of negative samples predicted to be positive.

Micro-F1 is the weighted average of micro-precision and micro-recall. The larger the F1, the better the performance of the model. The calculation method is as follows:


(14) $$F\,1=\frac{2\times \mathrm{Pr}e\times R\,e}{\mathrm{Pr}e+R\,e}$$

### Development of Education Information Management System of Migrant Children

To fully grasp the number of migrant children and education-related information, a migrant children management module is developed based on Shenyang children information management client and big data platform. The data in the *Integrated Management System of Migrant Population and Rental Housing* of the community grid office, the *Student Status Information Management System* of the education department, and *the Student Information Management System* of the primary schools in Shenyang city are integrated for comparison and analysis to develop the *Education Information Management System of Migrant Children*.

The system includes four functional modules: data comparison, classification management, data statistics, and data export. The data comparison module uses the large integrated management data of the community grid office and the student information of the education department to obtain the data for the children of primary school. After such data are imported into the system, a code is generated automatically; and the automatic matching of information is completed by selecting one or more information personal items (including name, gender, date of birth, father’s name, and mother’s name) and one or more position information items (this region, city, and province). The data information is classified into “migrant children” and “local children” with the ALGCNN model constructed in section “Definitions of Related Concepts” after the data comparison is completed. Data statistics refers to collect the children’s age, regional division, school status, school results, and class teacher evaluations. Finally, children’s information, academic performance tables, and learning assessment without query results are exported in the form of Excel.

### Statistical Processing and Analysis

SPSS22.0 software is utilized for data entry and analysis, mainly for descriptive statistical analysis of collected data, independent sample *t*-test, *F*-test, and correlation analysis. When *p* < 0.05, the difference is considered statistically significant.

## Results and Discussion

### Simulation Verification of ALGCNN Model

**FIGURE 3:**
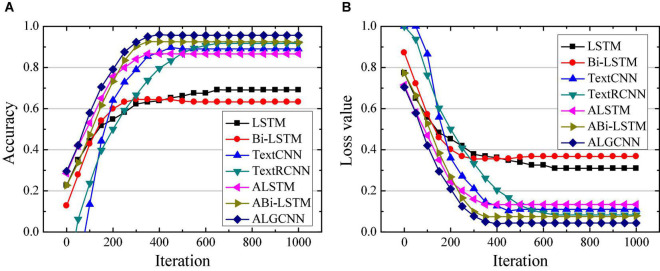
Comparison of classification performance of different models on the MR data set. **(A)** Is the comparison on classification accuracy; **(B)** is the comparison of classification loss value.

**FIGURE 4:**
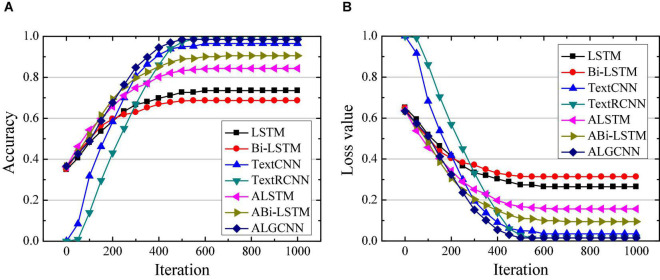
Comparison of classification performance of different models on the SST data set. **(A)** Is the comparison on classification accuracy; **(B)** is the comparison of classification loss value.

**FIGURE 5:**
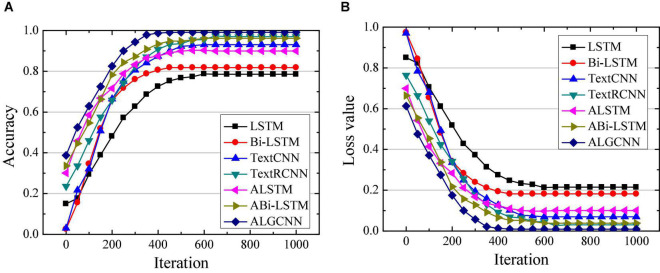
Comparison of classification performance of different models on the MPQA data set. **(A)** Is the comparison on classification accuracy; **(B)** is the comparison of classification loss value.

Simulation of ALGCNN model is verified using deep learning framework Tensorflow under Google open source, which integrates CNN, RNN, LSTM, and GRU models (Perez, 2019). The operating system for ALGCNN model simulation is Ubuntu 14.05, the development language is Python 3.6, and the framework used is Tensorflow 1.4.0. First, classification of the model is verified with MR, SST, and MPQA data sets. For MR data set, the text category is 2, the average sentence length is 20, the training set contains 9,595 words, and the testing set contains 1,050 words. For the SST data set, the text category is 5, the average sentence length is 18, the training set contains 9,845 words, and the testing set contains 2,010 words. For the MPOA data set, the text category is 2, the average sentence length is 3, the training set contains 9,500 words, and the testing set contains 1,105 words. The classification effects of LSTM (Chen et al., 2020), Bi-LSTM (Ye et al., 2019), Text CNN (Xie et al., 2020), Text RCNN (Huang et al., 2021), attention-based LSTM (ALSTM) (Liu et al., 2018), and attention-based Bi-LSTM (ABi-LSTM) (Song et al., 2018) are compared. The number of hidden layer neurons in the LSTM, Bi-LSTM, GRU, and RCNN model is set to 128, the text batch size is set to 100, the convolution kernel size in the CNN model is set to 3*3, and the number of convolution kernels is set to 128. The dimension of the attention layer is set to 128, the initial learning rate is set to 0.001, and the number of circuit training is 50. The differences in accuracy and loss values of different data sets are compared and analyzed in the text classification by each model, and the results are shown in Figures 3–5, respectively.

Figures 3A,B illustrate that the ALGCNN model shows the highest classification accuracy and the lowest loss value on the MR data set. There is no great difference in the classification accuracy and loss value between the ALSTM model and the ALGCNN model. The LSTM and Bi-LSTM models show the lowest classification accuracy and the largest loss values. The differences in the classification accuracy and the loss value of TextCNN, TextRCNN, and ABi-LSTM models are not obvious. At the same time, the constructed ALGCNN model has the fastest convergence speed.

Figures 4A,B suggest that the constructed ALGCNN model has the highest classification accuracy and the lowest loss value on the SST data set, followed by the ALSTM model, and the third is the TextRCNN model. The classification accuracy is the lowest and the loss is the largest for both LSTM model and Bi-LSTM model. In addition, the ALGCNN model shows the fastest convergence speed and lower probability of overfitting.

In Figures 5A,B, the constructed ALGCNN model shows the highest classification accuracy and the lowest loss value on the MPQA data set, followed by the ALSTM model, and the classification effects of the LSTM model and the ALGCNN model are not different so much. The LSTM and Bi-LSTM models show the lowest classification accuracy and the largest loss. At the same time, the ALGCNN model and the ALSTM model show the fastest convergence speed, and the ALGCNN model reduces the possibility of overfitting.

The difference in classification accuracy of different models on different data sets is quantitatively analyzed, and the results are shown in Table 1. Compared with the LSTM, Bi-LSTM, TextCNN, TextRCNN, ALSTM, and ABi-LSTM models, the classification accuracy of the ALGCNN model constructed on the MR data set is improved by 26.5, 32.3, 6.6, 3.9, 9.0, and 3.5%, respectively; the classification accuracy on the SST data set of the ALGCNN model constructed has increased by 25.0, 29.8, 2.0, 0.0, 14.2, and 8.0%, respectively; and the classification accuracy rate on the MPQA data set has increased by 20.6, 17.3, 6.0, 2.1, 9.2, and 2.9%, respectively.

**TABLE 1:** Text classification accuracy of different models on different data sets.

Model	MR (%)	SST (%)	MPQA (%)
LSTM	69.1	73.5	78.5
Bi-LSTM	63.3	68.7	81.8
TextCNN	89.0	96.5	93.1
TextRCNN	91.7	98.5	97.0
ALSTM	86.6	84.3	89.9
ABi-LSTM	92.1	90.5	96.2
ALGCNN	95.6	98.5	99.1

It is found that the classification accuracy of the ALSTM and ABi-LSTM models after the attention mechanism is added is significantly improved, which shows that the adding of the attention mechanism can improve the ability of the network model to extract features and then enhance the model’s ability to classify text. The classification performance of the TextCNN model is not good, which may be caused by the fact that the English TextCNN model does not consider the correlation between text word vectors (Banerjee et al., 2019). The ALGCNN model shows the best text classification effect, because the model first adopts LSTM and GRU models for feature screening, then applies the attention mechanism to increase the model’s ability to extract features, and finally obtains the final classification results through merging. Thus, it can improve the accuracy of text classification.

**FIGURE 6:**
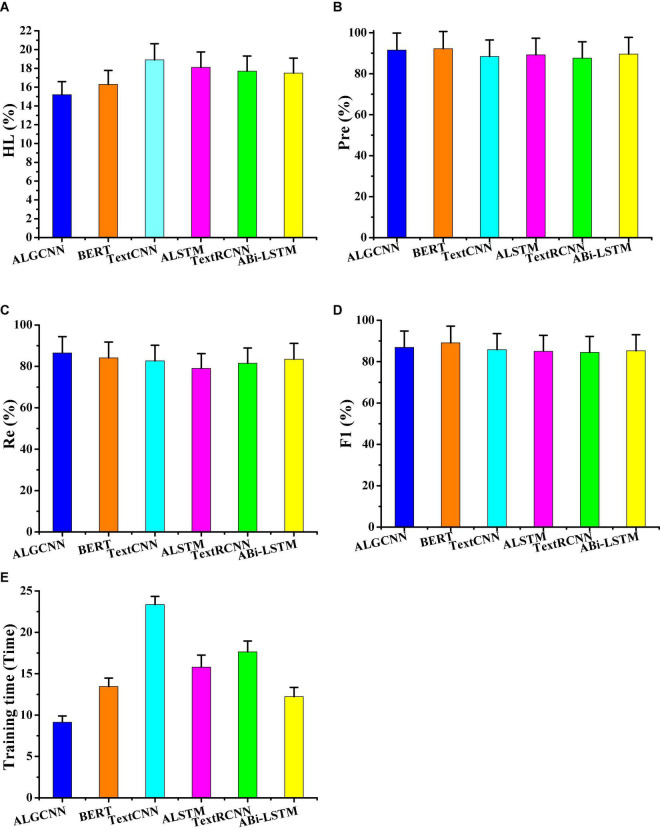
Comparison of evaluation index values of different models. **(A)** Showed the comparison of the HL value; **(B)** showed the comparison on Pre; **(C)** illustrated the comparison on the Re value; **(D)** was the comparison on F1; and **(E)** showed the comparison on training time.

Furthermore, the ALGCNN, ABi-LSTM, TextCNN, TextRCNN, ALSTM, and BERT model are compared regarding the average HL, Pre, Re, and F1s on different data set classifications (Figure 6). In different algorithms, ALGCNN shows the lowest HL (15.2 ± 1.38%), which is 1.1% lower than the BERT model. The *Pre* and F1 of the ALGCNN model are second only to the BERT model, and those of the *Pre* and F1 are 0.74 and 0.92% lower than the BERT model. The Re value of the ALGCNN model is higher than the highest among all models. The running time of the ALGCNN model is the shortest, which is 5.02 min shorter than that of the BERT model. Therefore, although the BERT model text classification accuracy rate and micro-F1 are better than the ALGCNN model, the training time of the ALGCNN model I is significantly shorter than that of the BERT model.

### Big Data Analysis of Migrant Children in Primary School of China

**FIGURE 7:**
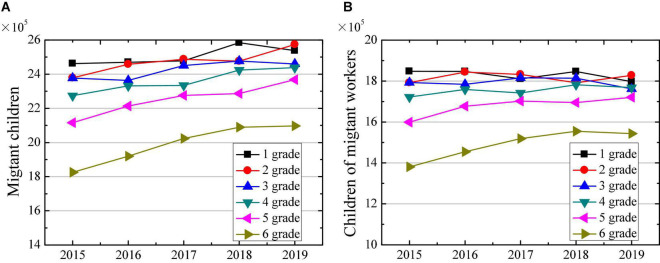
Changes in number of migrant children in primary school in 2015–2019. **(A)** Shows the changes in number of migrant children; and **(B)** shows the changes in number of children of migrant workers.

The data analysis results of children of migrant workers and children of migrant workers in primary school released by the Ministry of Education from 2015 to 2019 are shown in Figure 7. Figure 7A illustrates that the number of migrant children in different grades of primary school has shown a gradual increase from 2015 to 2019. In 2019, the number of migrant children in grade 1, 2, 3, 4, 5, and 6 increased by 0.76 × 10^5^, 1.95 × 10^5^, 0.83 × 10^5^, 1.65 × 10^5^, 2.52 × 10^5^, and 2.72 × 10^5^, respectively, compared with 2015. This may be because the younger generation of migrants is more inclined to keep their children around, so that the number of migrant children has shown an increasing trend year by year, which also points out that the number of left-behind children in rural areas is also gradually decreasing (Charania et al., 2019). Figure 7B reveals that the numbers of children of migrant workers in grades 5 and 6 have been increasing year by year, whereas the increase in the number of other grades has not changed much. The number of children of migrant workers in grades 1, 2, 3, 4, 5, and 6 in 2019 had increased by −0.50 × 10^5^, 0.36 × 10^5^, −0.31 × 10^5^, 0.46 × 10^5^, 1.21 × 10^5^, and 1.63 × 10^5^ people, respectively, compared with 2015.

**FIGURE 8:**
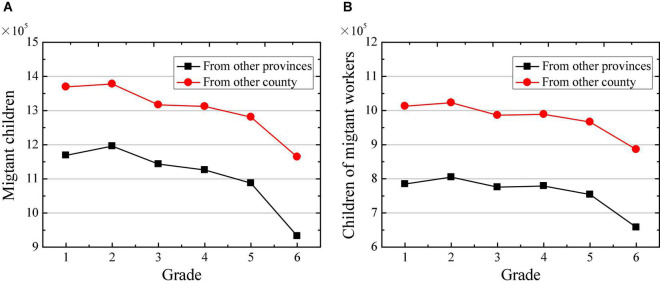
Changes in number of migrant children in primary school with different sources in 2019. **(A)** Shows the changes in number of migrant children; and **(B)** shows the changes in number of children of migrant workers.

Then, the changes in the number of migrant children with different sources in 2019 are compared. Figure 8A suggests that the number of migrant children from other counties in the province is significantly higher than the number of children from other provinces. With the increase of children’s learning grade, the numbers of children from different sources have shown gradual decrease, and the number of migrant children in grade 6 is significantly lower than that of other grades. Figure 8B indicated that among the children of migrant workers, the number of migrants from other counties in the province is also significantly higher than the number of migrants from other provinces. At the same time, with the increase of children’s learning grade, the number of children from different sources of immigration also discloses a gradual decrease. The number of migrant children in the grade 6 is significantly lower than other grades.

**FIGURE 9:**
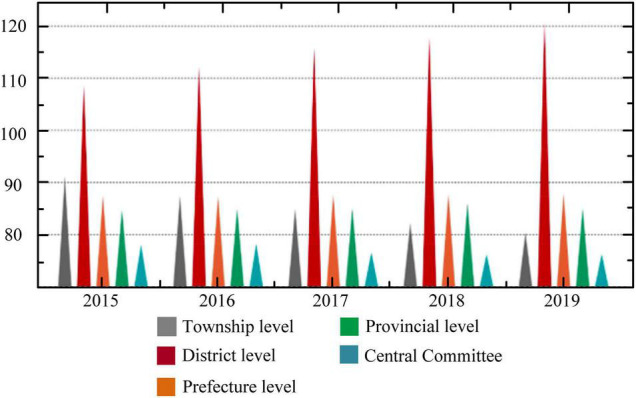
The proportion of government education expenditures at all levels in 2015–2019.

The educational expenditure data published by the *China Financial Yearbook* are applied to compare the differences and change trends in the proportion of education expenditures undertaken by governments at all levels in China from 2015 to 2019. Figure 9 indicates that the district- and county-level government undertakes the largest proportion in different years. The central government assumes the smallest proportion. With the gradual increase in years, the proportion undertaken by the township-level government has shown a gradual decrease, whereas the proportion of county-level government has shown a gradual increase. Therefore, it is speculated that the education issue of migrant children is also a fiscal issue. At present, compulsory education still suffers from many problems such as lack of national education funds, low proportion of central fiscal expenditures, uneven distribution of central fiscal transfer payments, unfulfilled “provincial overall planning” policy of compulsory education funds, and insufficient public finance investment in private education.

### Classification and Analysis on Information of Migrant Children

The school state and information of a total of 1,579 children in primary school are matched and classified from September 2018 to March 2019 in Shenyang city in the next semester, and the classification results of migrant workers’ children and local children are shown in Table 2. The accuracy of the constructed model is higher than 98% in both the classification of migrant children and local children.

**TABLE 2:** Classification results based on ALGCNN model.

ALGCNN model	Migrant children (*n* = 517)	Local children (*n* = 1062)
Accuracy (%)	510 (98.6)	1,048 (98.9)

First, the differences between migrant children and local children in different grades and gender distribution are compared and analyzed in Table 3. There is no significant difference in distribution of migrant children and local children in grades 3, 4, 5, and 6, and it is the same case in gender (*p* > 0.05). However, the proportion of migrant children in grades 1 and 2 is significantly higher than that of local children (*p* < 0.05). The high proportions of migrant children in the grades 1 and 2 may be because the children are so young that the parents want to take them and care them themselves (Hu and Wu, 2020).

**TABLE 3:** Comparison on distribution of migrant children.

Item	Subitem	Migrant children (*n* = 517)	Local children (*n* = 1062)	*p*-value
Grade	Grade 1	135 (26.1)	177 (16.7)	0.024
	Grade 2	110 (21.3)	180 (16.9)	0.031
	Grade 3	95 (18.4)	191 (18.0)	0.548
	Grade 4	90 (17.4)	173 (16.3)	0.872
	Grade 5	87 (16.8)	186 (17.5)	0.715
	Grade 6	71 (13.7)	155 (14.6)	0.772
Gender	Boy	322 (62.3)	748 (70.4)	0.127
	Girl	195 (37.7)	314 (29.6)	0.102

**FIGURE 10:**
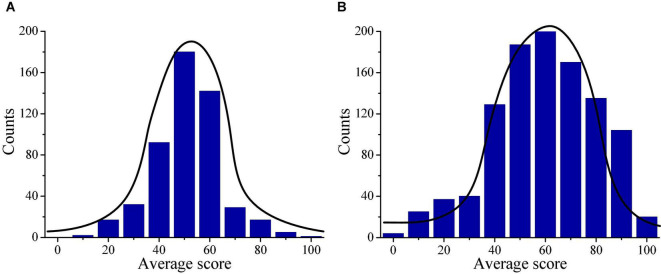
Descriptive statistics on final examination scores of children. **(A)** Illustrates the score distribution of migrant children; and **(B)** illustrates the score distribution of local children.

Then, the differences in the final examination scores and average scores of migrant children and local children are analyzed and compared, and the distributions are shown in Figure 10. Figure 10A reveals that the final average scores of migrant children are mainly distributed between 40 and 60 points, which may be because that it takes time for children to adapt to the learning environment in new schools. This may be the main reason for the poor performance of migrant children. Figure 10B that the final average scores of local children are mainly distributed between 40 and 90 points, which is in line with the actual situation of children’s score distribution.

**FIGURE 11:**
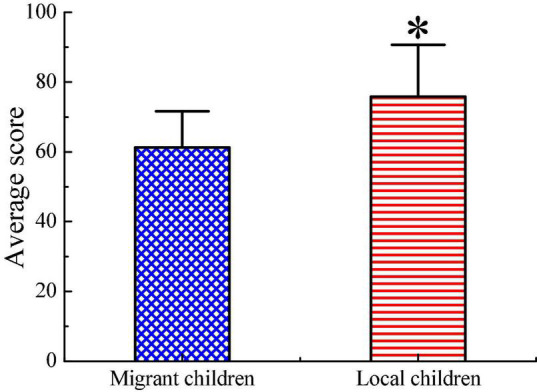
Comparison on average final examination score of children. ^∗^ Indicates that the difference between two groups is obvious (*p* < 0.05).

Third, the difference in the average final examination scores of migrant children and local children is illustrated in Figure 11. The average final examination score of local children is significantly higher than that of migrant children (*p* < 0.05).

**FIGURE 12:**
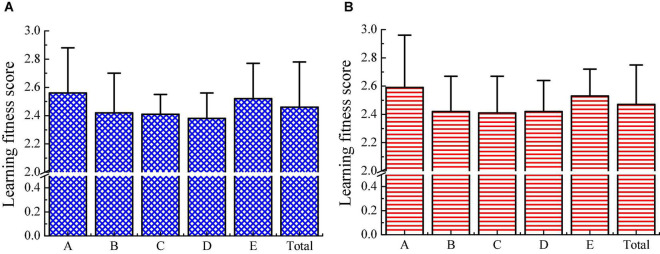
Comparison in children’s learning adaptability scores. **(A)** The scores of migrant children; **(B)** the score of local children; A refers to learning attitude; B refers to listening method; C refers to learning skills; D indicates the adaptability to the school teaching environment; and E represents the independent learning ability.

The learning adaptability scores of migrant children and local children are analyzed. Figures 12A,B show that both migrant children and local children have lower scores in listening methods, learning skills, and adaptability to learning and teaching environment, whereas both have higher scores in terms of learning attitude and independent learning ability. After comparison, it is found that the differences are not obvious in each dimension score and total score between migrant children and local children (*p* > 0.05). Migrant children have a weak ability to adapt to the teacher’s teaching methods and the teaching schedule, which indicates that migrant children need to improve their listening methods during the learning process.

## Conclusion

This research aims to explore the impact of China’s existing education policies on the educational integration of migrant children. First, ALGCNN model is constructed for text classification of children’s education information. After analysis of its performance, it is applied to text classification of migrant children and local children, and the influencing factors of migrant children’s education integration are discussed. The results show that ALGCNN model has a high text classification accuracy, and children’s education cost, way of attending classes, learning skills, and adaptability to learning environment are the main influencing factors for educational integration of migrant children. However, there are still some shortcomings in this study. This study only collects big data to conduct a comparative analysis on the education status of migrant children, but does not conduct a correlation regression analysis on the influencing factors of different dimensions of migrant children’s education integration and education policies. In the follow-up, based on the results and many field investigations, further regression analysis will be made on influencing factors of different dimensions of children’s educational integration and education policies, and reasonable suggestions will be given based on existing problems.

In terms of practical issues, it is also necessary to provide provisions and protection in the legislative field, especially for large immigrant provinces such as Hainan, Guangdong, Zhejiang, and so on. It is necessary to share information in the source provinces of local immigrants, establish and perfect local legislative guarantee mechanism, and provide legal support for improving education and fill in legislative vacancies.

In short, the results of this article can provide a theoretical basis for solving the education problems of migrant children and improving the education policies of migrant children.

## Data Availability Statement

The raw data supporting the conclusions of this article will be made available by the authors, without undue reservation.

## Ethics Statement

The studies involving human participants were reviewed and approved by the Ethics Committee of Northeastern University. The patients/participants provided their written informed consent to participate in this study. Written informed consent was obtained from the individual(s) for the publication of any potentially identifiable images or data included in this article.

## Author Contributions

All authors listed have made a substantial, direct, and intellectual contribution to the work, and approved it for publication.

## Conflict of Interest

The authors declare that the research was conducted in the absence of any commercial or financial relationships that could be construed as a potential conflict of interest.

## Publisher’s Note

All claims expressed in this article are solely those of the authors and do not necessarily represent those of their affiliated organizations, or those of the publisher, the editors and the reviewers. Any product that may be evaluated in this article, or claim that may be made by its manufacturer, is not guaranteed or endorsed by the publisher.
